# Effect of Extraction Temperature on Pressurized Liquid Extraction of Bioactive Compounds from *Fucus vesiculosus*

**DOI:** 10.3390/md20040263

**Published:** 2022-04-12

**Authors:** Adane Tilahun Getachew, Susan Løvstad Holdt, Anne Strunge Meyer, Charlotte Jacobsen

**Affiliations:** 1 Research Group for Bioactives-Analysis and Application, National Food Institute, Technical University of Denmark, 2800 Kongens Lyngby, Denmark; atige@food.dtu.dk (A.T.G.); suho@food.dtu.dk (S.L.H.); 2 Department of Biotechnology and Biomedicine, DTU Bioengineering, Technical University of Denmark, Søltofts Plads 221, 2800 Kongens Lyngby, Denmark; asme@dtu.dk

**Keywords:** *Fucus vesiculosus*, low polarity water, antioxidant activity, crude fucoidan, marine phenolic compounds

## Abstract

This study was aimed at investigating the effect of low polarity water (LPW) on the extraction of bioactive compounds from *Fucus vesiculosus* and to examine the influence of temperature on the extraction yield, total phenolic content, crude alginate, fucoidan content, and antioxidant activity. The extractions were performed at the temperature range of 120–200 °C with 10 °C increments, and the extraction yield increased linearly with the increasing extraction temperature, with the highest yields at 170–200 °C and with the maximum extraction yield (25.99 ± 2.22%) at 190 °C. The total phenolic content also increased with increasing temperature. The extracts showed a high antioxidant activity, measured with DPPH (2,2-Diphenyl-1-picrylhydrazyl) radicals scavenging and metal-chelating activities of 0.14 mg/mL and 1.39 mg/mL, respectively. The highest yield of alginate and crude fucoidan were found at 140 °C and 160 °C, respectively. The alginate and crude fucoidan contents of the extract were 2.13% and 22.3%, respectively. This study showed that the extraction of bioactive compounds from seaweed could be selectively maximized by controlling the polarity of an environmentally friendly solvent.

## 1. Introduction

The marine environment is endowed with a variety of untapped resources, which could be exploited for the production of bioactive compounds used for foods, pharmaceuticals, and cosmetics. Among marine organisms, brown seaweeds are known to contain different kinds of bioactive compounds such as pigments, proteins, phenolic compounds, and omega-3 fatty acids [1]. The *Fucus vesiculosus* Linnaeus (1753), popularly known as bladder wrack, is an edible brown seaweed that is widely distributed in the intertidal areas of many cold and warm temperate regions in the Northern Hemisphere, including the western Baltic Sea, the Atlantic coasts of North America and Europe, and the western Mediterranean. *Fucus vesiculosus* consists of a holdfast, a small stipe, and flattened dichotomously-branched blades with several pairwise air bladders, which keep them afloat in a vertical position when submerged [2,3]. *Fucus vesiculosus* contains several interesting bioactive compounds such as phenolic compounds, protein, carotenoid pigments such as fucoxanthin, and polysaccharides such as alginate and fucose, containing a sulfated polysaccharide known as fucoidan. Due to the presence of these compounds, this seaweed has been used for food and feed in Europe since the 17th century, as well as in traditional medicine in China [3]. The main bioactive compounds in *F. vesiculosus* are phenolic compounds (phlorotannins) and polysaccharides (fucoidan). Studies reported that this brown seaweed accumulates phlorotannins up to 12% of its dry weight depending on the harvest season, geographic origin, solar exposure, and salinity of the water [1]. Phlorotannins are complex phenolic compounds found in brown seaweeds. They are oligomers of phloroglucinol (1,3,5-trihydroxy benzene) monomer units. Phlorotannins’ primary role in brown seaweeds is to provide a protective or defense system for the cell from the environment (e.g., sun) and herbivores. They also have several biological activities such as antioxidant, anticoagulant, anticancer, and antidiabetic ones [4].

Fucoidan is a sulfated polysaccharide with a basic structure comprised of a sulfated fucose backbone, but it also contains small quantities of other sugars, such as xylose, uronic acids, and galactose [5]. The composition and structure of fucoidans depend on the geographic origin, the harvest/extraction season, species type, growth stage, and extraction method [6]. The molecular size of fucoidan has been reported to vary significantly among the different species and extraction methods. Different molecular sizes, from the smallest sizes of 43 kDa to large sizes up to 1600 kDa, have been reported [5]. Fucoidans have been reported to possess several biological activities such as antioxidant [7], anticoagulant [8], antitumor [9], anti-inflammatory [10], and neuroprotective ones [11].

The extraction of bioactive compounds from seaweed is mainly performed using traditional solvent extraction (SLE). This extraction method has several drawbacks including the use of a large amount of organic solvent, acids, and alkaline, a long extraction time, and selectivity problems. Moreover, since SLE is conducted in an open system, the important compounds are exposed to oxidation. To mitigate these problems, several studies have reported the application of emerging green extraction technologies for the extraction of bioactive compounds from different seaweed species. These technologies include ultrasound-assisted extraction [12], microwave-assisted extraction [13], enzyme-assisted extraction [14], pressurized liquid extraction [15], natural deep eutectic solvents [16,17] and supercritical carbon dioxide extraction [18]. Among these green extraction technologies, pressurized liquid extraction, also known as subcritical water extraction or low polarity water (LPW) extraction (LPWE), has drawn considerable attention from researchers due to the extraordinary physical properties of LPW. LPW is kept between its boiling point (100 °C) and critical temperature (374 °C), at a sufficiently high pressure to keep it in liquid form. Water at these conditions has a low dielectric constant, low viscosity, high dissociation constant (*Kw*), and low surface tension, which are comparable to some common organic solvents, such as ethanol and methanol. The polarity of water can be tuned by changing the extraction temperature. For instance, increasing the temperature from room temperature to 200 °C could reduce the dielectric constant from 88 to 33, similar to that of organic solvents such as ethanol and methanol [19]. In addition, by increasing the temperature, the viscosity and surface tension of the water decrease while the diffusivity increases, allowing deep penetration of the solvent into the matrix, thus enhancing the efficiency and speed of the extraction process [19,20]. So far, some attempts have been made to extract bioactive compounds from several seaweed species, including red seaweed [21,22], green seaweeds [23], and several brown seaweeds [24,25]. However, to the best of our knowledge, there is no reported study on the use of LPW and the influence of the extraction temperature on the extraction of bioactive compounds from the Nordic seaweed *F. vesiclulosus*. Therefore, this study aimed to investigate the influence of LPW on the extraction of bioactive compounds from Nordic seaweed *F. vesiclulosus*. Therefore, the influence of the different extraction temperatures on the extraction yield, total phenolic content (TPC), crude alginate and fucoidan content, and antioxidant activity from *F. vesiculosus* using LPWE was studied. The results were evaluated using a correlation matrix.

## 2. Results and Discussion

### 2.1. Extraction Yield

**Figure 1:**
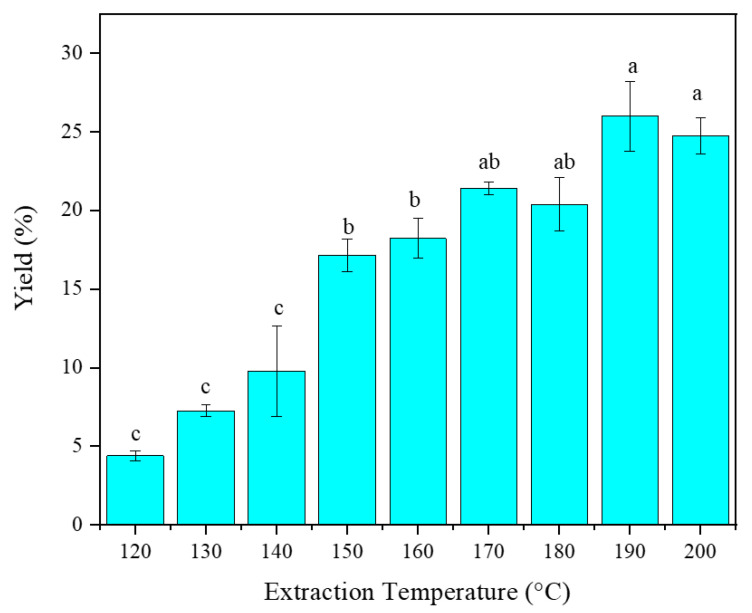
Extraction yield at different temperatures starting from 120 °C to 200 °C of *Fucus vesiculosus*, using LPW extraction. Different letters indicate significant differences between the different extraction temperatures.

The extraction yield is shown in Figure 1. The extraction yield increased with increasing the extraction temperature, with the highest yields found at the temperatures from 170–200 °C with no significant difference between the yields at these temperatures, but with the maximum at 190 °C with a 25.99 ± 2.22% extraction yield. The effect of temperature above 200 °C could not be evaluated, due to the instrument’s maximum temperature limitations. The reason for increasing yield could be associated with the increase in the hydrolysis of the seaweed with increasing temperature. Reports from several studies demonstrated that temperature is an important factor involving pressurized liquids. Increasing temperature disrupts matrix interactions between analytes and samples, which are caused by hydrogen bonding, van der Waals forces, and dipole interactions. This facilitates deeper penetration of the solvent into the sample matrix [26]. Furthermore, increasing temperature decreases the viscosity of the solvent, which consequently improves the penetration of the solvent into the sample matrix. This phenomenon increases the diffusion of the analyte into the solvent medium and improves the extraction yield [27]. Ibañez et al. [28] also reported that increasing temperature could facilitate the extraction yield, by increasing both the solubility and mass transfer rates. However, very high temperatures could have a negative impact on the yield. At very high temperatures, the produced hydrolysates could be further decomposed into smaller molecules or organic acids that consequently decrease the extraction yield [29]. Nevertheless, temperature has a significant effect on the extraction yield.

### 2.2. Crude Alginate and Fucoidan Content

**Figure 2:**
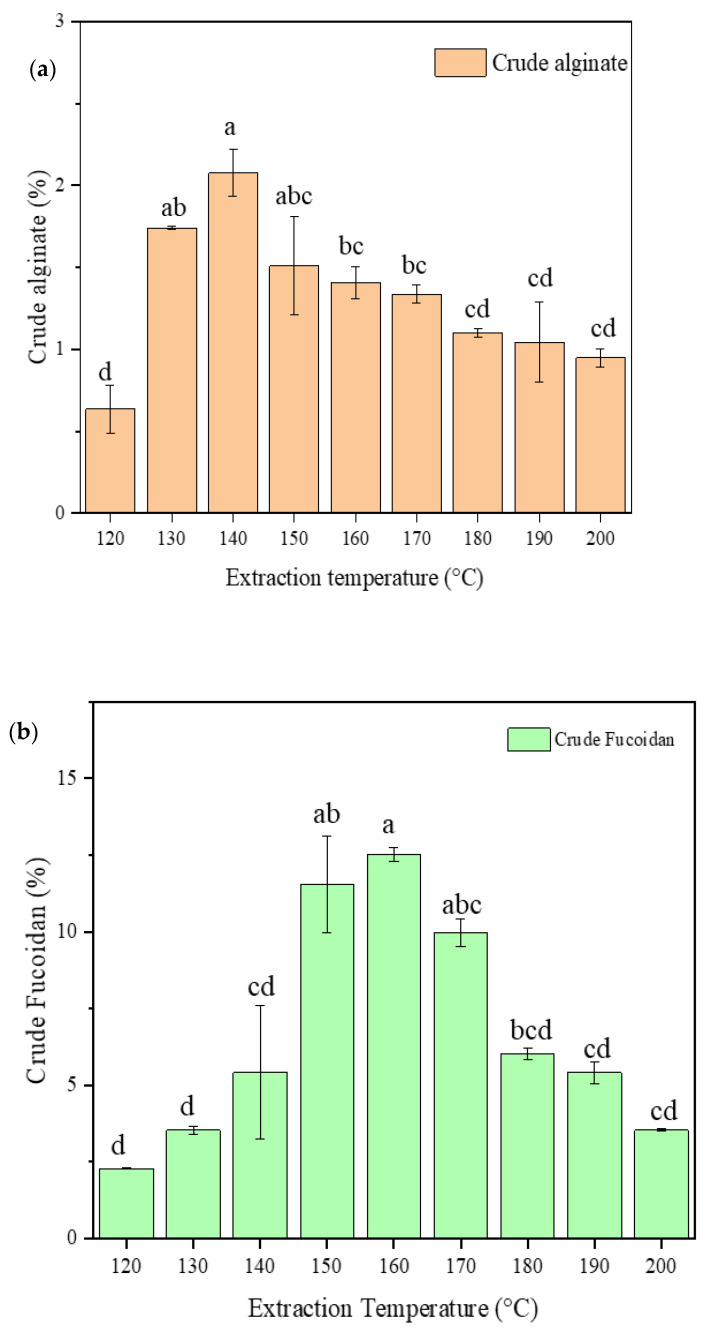
The crude alginate content (**a**) and crude fucoidan (**b**) content of the extracts. The alginate fucoidan content was represented as percentage of the dried *F. vesiculosus*. The different letters on the top of each bar indicate the significant differences (*p* < 0.05) among the different extraction temperatures.

Figure 2a,b show the crude alginate and fucoidan contents of the extract at different extraction temperatures. The alginate content of the extract increased between 120 °C to 130 °C. The 130–150 °C extractions were not significantly different, but with decreasing alginate yield at higher temperatures. The maximum alginate extracted was (2.08 ± 0.12%) at 140 °C. Alginates are polysaccharides, consisting essentially of d-mannuronic (M) and L-glucuronic (G) acid units linked with (1→4) bonds, arranged as homopolymeric (M or G) or heteropolymeric (MG) forms. In the presence of divalent cations, such as Ca^2+^, the G-blocks form “egg-box” junctions and facilitate the formation of gel between the two opposite chains [30]. However, when the temperature increases, it could lead to the depolymerization of the longer molecular size polymer into smaller groups, which may not interact with Ca^2+^ during the precipitation of alginate using CaCl_2_ from the extract solution. Furthermore, previous reports showed that *F. vesiculosus* alginates are very sensitive to high temperatures, and their polymeric structure drastically reduces even with a slight change in treatment temperatures [31]. Thus, the optimal extraction temperature for maximizing the yield of alginates is at about 130–150 °C.

Similar to alginate, the yield of crude fucoidan content also increased with increasing temperature with highest yield for fucoidan recorded at 150–170 °C with the maximum average recorded (12.52 ± 0.24%) at 160 °C. Hereafter, the yield started to decrease, reaching a low yield similar to that at 120 °C when the temperature was 200 °C. A similar trend has been reported on the extraction of fucoidan from *S. japonica*, using subcritical water extraction [24]. Increasing the temperature of LPW resulted in increasing the dissociation constant of water (*K_w_*). The increase in *K_w_* derives the formation of hydronium (H3O^+^) and hydroxide ion (HO^−^) in the reaction system [19]. Hydroxide ions and protons interfere with the hydrogen bonds between the different polysaccharides, and increase the extraction yield by releasing them into the solution [32]. The increasing temperature could also create a higher mass transfer rate and solvent diffusion, while lowering surface tension and viscosity made more polysaccharides dissolve in the extraction medium [12]. The reason for the decreasing of fucoidan content at higher temperatures could be explained by the degradation of the fucoidan extracted at the earlier stages of the extraction conditions. Different previous studies documented that a high extraction temperature or longer extraction time could affect the extraction yield of fucoidan from several brown seaweed species [33,34]. A slightly higher fucoidan yield, 13.56%, was reported by Saravana et al. [24] extracted from *S. japonica* using subcritical water at an optimized temperature of 127 °C. Rodríguez-Jasso et al. [35] reported 16.5% for *F. vesiculosus* extracted using an autohydrolysis technique at a temperature of 180 °C. Fucoidans are fucose-rich sulfated polysaccharides located at the fibrillar cell walls and intercellular spaces of brown seaweeds, so degrading the cell wall structure first will expose the intracellular fucoidans to diffuse into the extracting medium [33]. As described above, the alginate content started to decrease after 150 °C, indicating the degradation of the cell wall structure and exposing the intracellular fucoidan to be extracted by the LPW. Nevertheless, by tuning the temperature, the extraction conditions can be optimized to get the maximum yield of fucoidan, as demonstrated here.

### 2.3. Total Phenolic Content

**Figure 3:**
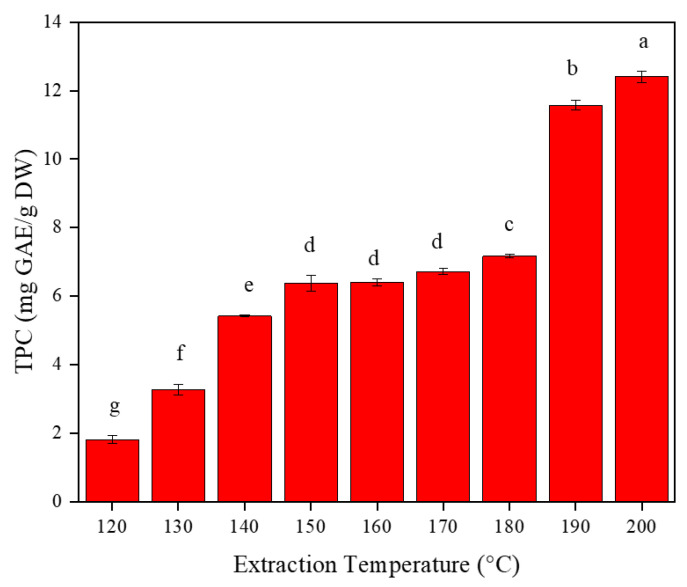
Total phenolic content (TPC) of the extracts at different extraction temperatures (120–200 °C). The different letters on the top the bars indicate the presence of significant differences (*p* < 0.05) among the different extraction temperatures.

The total phenolic content of the extracts is shown in Figure 3. TPC increases linearly, with an increasing extraction temperature in the range of 120–150 °C. In the temperature range 120–150 °C, the TPC increased by a factor of two with every 10 °C increment of temperature. However, the TPC started to decrease after a 190 °C extraction temperature. The reason for increasing the TPC of the extracts with temperature could be explained mainly by the change in the dielectric constant of water at higher temperatures, which subsequently decreases the polarity of water that is comparable to most common organic solvents. Several studies reported that high content of phenolic compounds can be extracted using fewer polar solvents than polar solvents [3]. Most phenolic compounds have lower solubility in water than in organic solvents, such as methanol or ethanol at ambient temperature. The reason for this is their differences in polarity. Water has a higher polarity than methanol and ethanol at room temperature. However, as in the case of this study, the polarity of water can be fine-tuned by controlling the temperature and pressure at subcritical conditions. The polarity of water could be equivalent with most of the organic solvents, such as ethanol and methanol at a temperature close to 200 °C. The high amount of TPC at high temperature could also be due to less inhibiting interaction between phenolic compounds and proteins during the extraction, or even by breaking hydrogen bonds between phenolic compounds-protein complexes [36]. Therefore, it is possible to modify the polarity of subcritical water by tuning the temperature and pressure, so that water can be used instead of environmentally unfriendly organic solvents to extract low polar components from *F. vesiculosus*, which are otherwise difficult to extract using water at ambient conditions. Different research works from Pangastuti et al. , Gereniu et al. [15] Saravana et al. [24], and many others on the extraction of phenolic compounds from seaweed all reported a similar trend. We have also conducted a conventional extraction of total phenolic compounds for comparison purposes, and the TPC was 36.9 ± 0.30 mg for GAE/g of dried seaweed. The extraction showed a higher result because the extraction was conducted for a total of 48 h. However, the values of conventional extraction are different from those reported by Obluchinskaya et al. [17] who reported 5–200 mg phloroglucinol per g of dry raw material (mg/g d.m.), for *F. vesiculosus* extracts obtained using several organic solvents that they used for their analysis. As mentioned, the TPC of algae is influenced by several abiotic and biotic factors such as the species, plant stage, size, age, reproductive status, location, depth, nutrient enrichment, salinity, light intensity exposure, ultraviolet radiation, intensity of herbivory, and time of collection. Therefore, the full exploitation of algal diversity and complexity requires knowledge of environmental impacts, and an understanding of biochemical and biological variability [36].

### 2.4. Phlorotannin Content

**Figure 4:**
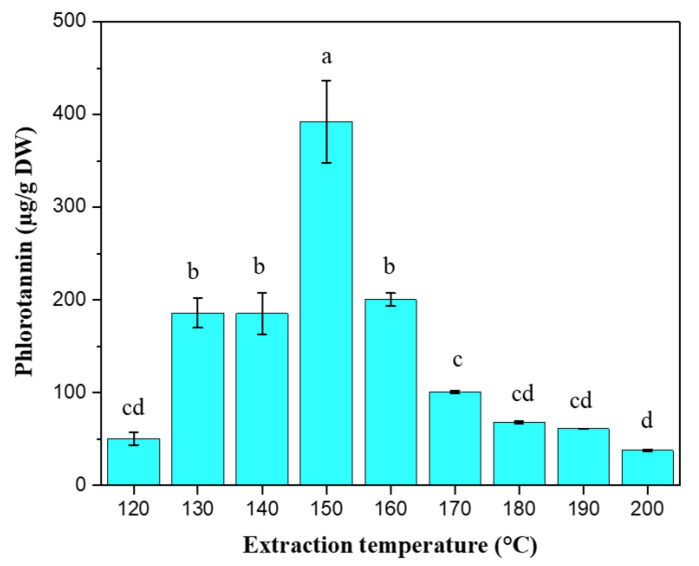
Phlorotannin contents of the extracts. The y-axis represents the phlorotannin content in terms of phloroglucinol equivalent (PGE). Bars with different small letters on top are significantly different (*p* < 0.05).

The phlorotannin content of the extracts is depicted in Figure 4. The highest and the lowest phlorotannin contents were registered for the extract obtained at 150 and 200 °C with 392.21 ± 44. 19 and 37.59 ± 1.05 µg PGE/g DW, respectively. After reaching its highest level at 150 °C, it starts to decrease beginning from 160 °C. There is no significant difference (*p* > 0.05) between low-temperature extracts (120 °C) and high-temperature extracts (170–200 °C). At low temperatures, the polarity of water is relatively high and the solubility of phlorotannin is low. This could be the reason for the low phlorotannin content at 120 °C. Even though the polarity of water decreases with increasing temperature, at high temperatures the thermal decomposition of the phlorotannin is high. Thus, this could the possible reason for its lower content in the temperature range of 160–200 °C. The reason for the decreasing trend with increasing temperature could be due to the thermal decomposition of phlorotannin [37]. Caterino et al. [3] reported higher values (2.92 ± 0.05 mg PGE/g DW) for *F. vesiculosus* extracted using aqueous acetone (67% *v*/*v*) and analyzed with the DMBA assay. Amarante et al. [38] also reported higher values (3.16 ± 0.06 mg PGE/g DW) for *F. vesiculosus* extracted using optimized microwave-assisted extraction conditions temperature of 75 °C and aqueous ethanol (57% *v*/*v*) as the extracting solvent. On the other hand, Ferreira et al. [37] reported lower values (0.226 mg PGE/g DW), as compared to the highest values found in this study, for the same seaweed species extracted at 120 °C. The observed differences in the phlorotannin content of the current and previous studies could be due to several factors, including the harvest season of the seaweed, the geographical location, the method, and the solvent used for the extraction.

### 2.5. Individual Phenolic Compounds

**Figure 5:**
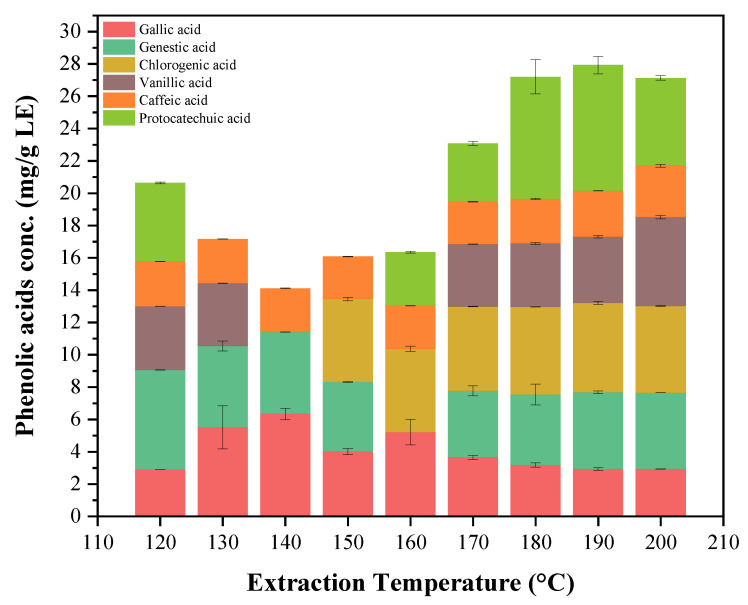
The concentration of the individual phenolic acid identified in each extract, obtained at different extraction temperatures (120–200 °C). The error bars indicate the standard deviations of at least two replicates.

The individual phenolic contents of the extract are depicted in Figure 5. Six different kinds of phenolic compounds were detected in the extracts. The amount of the phenolic acids varied from 3.0 mg/g LE–7.80 mg/g LE. Both the type and amount of the phenolic acids varied considerably with the extraction temperature. Caffeic and gallic acids were detected in all extraction conditions, with caffeic acid having a relatively constant amount in all extracts, whereas gallic acid showed the highest content in the extract obtained at 140 °C. Chlorogenic acid was not detected in the extracts obtained at lower temperatures, however, it was detected in all extracts starting from an extraction temperature of 150 °C. This might be due to the solubility of chlorogenic acid in water at a higher temperature. Similarly, protocatechuic and vanillic acids were not detected in the temperature range 130–160 °C, which could be due to their low solubility at the relatively high polarity of water in the temperature range 130–160 °C [39]. The polarity of water significantly changes at a higher temperature. The polarity can be measured in terms of dielectric constant, which means a high dielectric constant corresponds to a high polarity and vice versa. The dielectric constant of water decreased by more than half between room temperature (25 °C and 200 °C), which is from 80–37. At higher temperatures, the dielectric constant of water decreases, as does the polarity of water, making water behave similar to those of organic solvents such as ethanol or methanol. All the six phenolic acids found in the current study have, also, been reported by Farvin and Jacobsen [40] in the water extracts of *F. vesiculosus* using the conventional solvent extraction method. However, except for gallic and gentisic acids, the concentration of all other phenolic acids in the current study was found to be higher than those reported by Farvin and Jacobsen [40]. In a recent study, Sanches-Bonet et al. [41] have reported 12 different kinds of phenolic acids. The phenolic acids detected by their study, but not in our current study, include coumaric acid, catechin, epicatechin, rutin, and ferulic acid. The reason for the observed differences could be due to several conditions, such as the geographic location, the extraction and analysis methods, the harvest season, and the age of the tissue [30,42].

### 2.6. Antioxidant Activity

#### 2.6.1. DPPH Radicals Scavenging Activity

The antioxidant activity of *F. vesiculosus*, as measured by DPPH radicals scavenging activities, is shown in Table 1. The antioxidant activity was expressed in IC_50_ values, meaning that the lower the IC_50_ value was, the higher the antioxidant activity. The highest antioxidant activity registered in this study was by the extracts obtained at 160 °C with 0.14 mg DE/mL, but it was only significantly different from the lowest DPPH recorded for the extract recovered at 120 °C with 0.23 mg/mL. Different values of DPPH radical scavenging activities have been reported by several researchers. André et al. [43] reported a lower DPPH value for water extracts of *F. vesiculosus* obtained at 100 °C. The current result is higher than each of the 10 different brown seaweed species from the Brittany coasts, extracted using dichloromethane and methanol (1:1, *v*/*v*), as reported by Zubia et al. [44]. Similarly, both Agregán et al. [45] and Agregán et al. [46] reported a lower DPPH radical scavenging activity for *F. vesiculosus* extracts extracted using water and ethanoic water (50:50, *v*/*v*), respectively. However, Farvin and Jacobsen [40] and Wang et al. [47] reported higher DPPH values (IC_50_ values between of 8.3 and 10.7 µg/mL) for water and 70% acetone extracts of the same seaweed species, respectively. The observed differences in DPPH scavenging values could be attributed to several factors, including the type of extraction method, the seaweed species, the harvesting season, and the locations. Reactive oxygen species are responsible for the initiation of lipid oxidation in food systems, pharmaceuticals, cosmetic products, and, even, in a living system. In this regard, the high radical scavenging activity of the extract could contribute to replacing synthetic antioxidants.

#### 2.6.2. Metal-Chelating Activity

The metal-chelating activity of the extracts is shown in Table 1. The highest and lowest MC activity were found at 130 °C and 200 °C with IC_50_ values of 1.39 mg/mL and 23.05 mg/mL, respectively, with the highest being significantly different from 180–200 °C. High metal-binding activities of polysaccharides from seaweeds such as alginate, fucoidan, agar, and carrageenan have been reported. However, at high temperatures, the big molecular size of polysaccharides has already been degraded into smaller sugar molecules, and there will not be enough metal-chelating species left in the extract. This might be the reason for the observed low MC activity with increasing temperature. The current results are lower than those reported by Sumampouw et al. [48] who reported 1.1 mg/mL for *F. vesiculosus*, obtained at optimum extraction conditions using PLE. On the other hand, Wang et al. [47] reported 95% of metal-chelation activity for water extracts of *F. vesiculosus*, at a concentration of 5 mg/mL. In addition, Wang et al. [49] reported that *F. vesiculosus* extracts are not good metal-chelating agents, rather they are potent free radical scavengers and primarily of chain-breaking antioxidants. The antioxidant activity of the seaweed extracts depends on the content of the free radical scavenging species and metal-chelating species present in the extract. Conversely, the content of these antioxidants depends on, among other things, the harvest season seaweed, the extraction method, and the solvent. Thus, there is no common comparison ground to reach a conclusion on the variations of the different bioactivities.

### 2.7. Correlation Matrix

**Figure 6:**
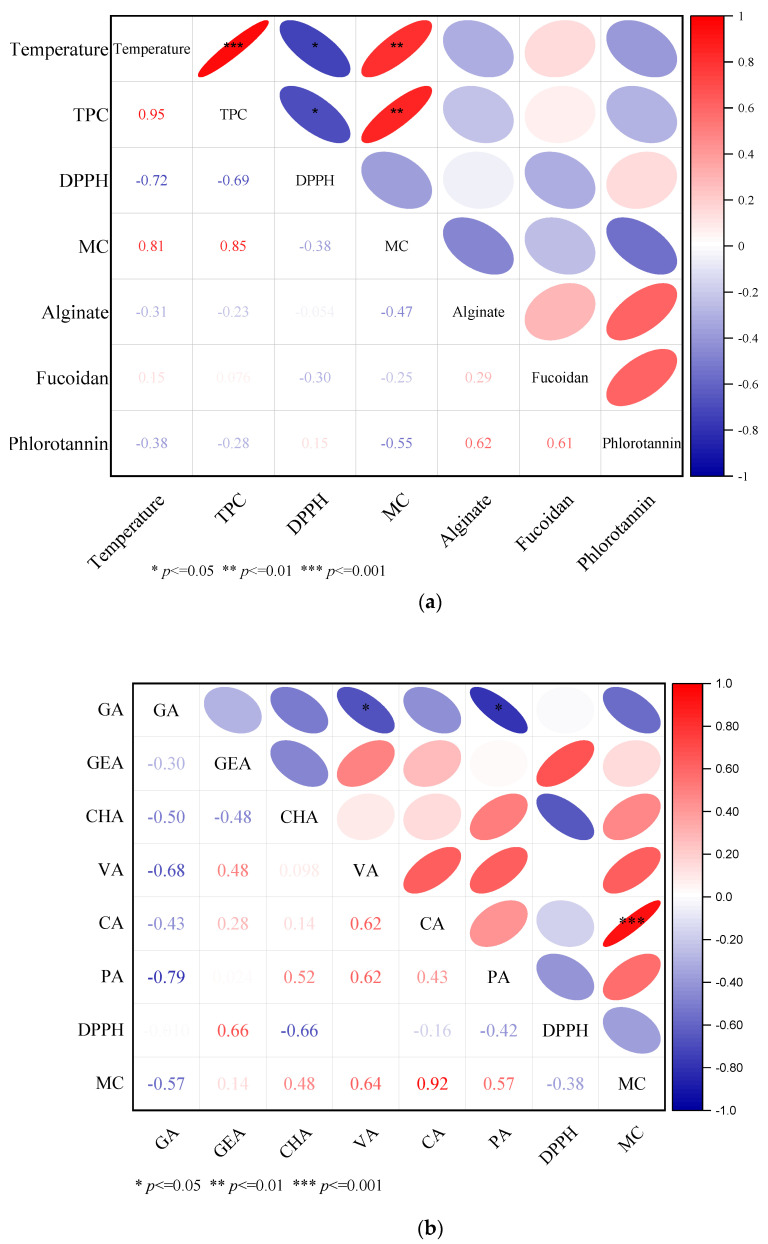
(**a**). Correlation plot indicating interaction among temperature, TPC, alginate and fucoidan contents, DPPH radical scavenging activity, and metal-chelating (MC) activity. The intensity of the color shows the strength of the correlation, with the red and blue colors for positive and negative correlations, respectively. The asterisks on the ellipses of the upper triangular matrix show the significance of the correlation at different *p*-values. The different numbers on the lower triangular matrix shows the correlation coefficient (r-values). The intensity of the font color of the r-values fades with a decrease in the r-values. (**b**) shows the correlations among the different phenolic acids (GA-gallic acid, GEA-genestic acid, CHA-chlorogenic acid, VA-vannilic acid, CA-caffeic acid, PA-protocatechuic acid) and the DPPH and MC values.

To understand the interaction among the extraction temperatures, TPC, alginate content, fucoidan content, and antioxidant activity, a Pearson correlation was calculated on the mean values of each parameter. Figure 6b shows the correlation plot, and the color intensity on the left side of the graph indicates the strength of the correlation. The darker the red color is, the stronger the positive correlation, and the darker the blue color indicates a stronger negative correlation. The asterisks on the ellipse show the *p*-values of the significant correlations. The TPC showed strong significant positive correlation with temperature (r = 0.95, *p* ≤ 0.001). However, there was no significant correlation between extraction temperature and both alginate and fucoidan contents (*p* > 0.05). The DPPH radical scavenging activity showed a significant negative correlation (r = −0.72, *p* ≤ 0.05) with temperature and TPC (r = −0.69, *p* ≤ 0.05). The negative correlation of DPPH radical scavenging activity with TPC is in agreement with previous studies by Silva et al. [50], Sánchez-Bonet et al. [41], Farvin and Jacobsen [40], and Balboa et al. [42]. However, it is in disagreement with other previous studies such as Díaz-Rubio et al. [51], Agregán et al. [45], and Jimenez-Escrig et al. [52]. This result may indicate that other co-extracted compounds such as peptides, protein, and polysaccharides contributed to the radical scavenging activity of the extracts [53]. The MC showed a strong positive correlation with extraction temperature (r = 0.81, *p* ≤ 0.01) and with TPC (r = 0.85, *p* ≤ 0.05). The TPC analysis method based on FC reagent is influenced by the presence of other reducing agents such as proteins and sugars [54]. Thus, the positive correlation between MC and TPC may not be due to the high level of TPC, but rather be due to other reducing agents. Moreover, reducing agents such as protein and peptides have long been reported to possess metal-chelating activities [55,56,57,58]. Furthermore, the negative correlation (r = −0.28) between TPC and phlorotannin content of the extracts supports the argument that the TPC analysis based on the FCR method is influenced by other reducing agents and leads to an overestimation of the TPC values. There is a weak positive correlation (r = 0.15) between DPPH and phlorotannin content, indicating the contribution of phlorotannin to the DPPH radical scavenging activity of the extracts.

To further investigate how TPC is correlated positively with MC, and which specific phenolic acid is responsible for the MC activity of the extract, Pearson correlation coefficients were calculated on each phenolic acid, DPPH, and MC activity. Figure 6b shows the correlation plots. As shown in Figure 6, the MC is positively correlated with all phenolic acids, except with gallic acid. However, only the correlation between caffeic acid and MC is positive and very strong (r = 0.92, *p* ≤ 0.001). Several studies reported that caffeic acid is a very strong metal chelator [59,60,61,62]. Sørensen et al. [63] reported the highest MC activity of caffeic acid as compared with other hydrocinnamic acids, such as ferulic and coumaric acid. Caffeic acid is a hydrocinnamic acid with an *o*-hydroxyl (catechol) group. This group is a preferred binding site for metals and leads a strong chelating activity for caffeic acid [64]. Different studies reported a positive correlation between several phenolic acids, such as caffeic and chlorogenic acids, and DPPH radical scavenging activities. However, in the current study, DPPH only correlated positively with the content of genestic acid (r = 0.66, *p* ≤ 0.05) (Table S1). To explain the origin of this contradictory finding as well as to understand and identify which classes of compounds are responsible for the metal-chelating and radical scavenging activity, further studies on purification and fractionation of the crude extract are required.

## 3. Materials and Methods

### 3.1. Sample Preparation

Brown seaweed *F. vesiculosus* was harvested in May 2020 from Bellevue Beach, (55°46′17.4″ N 12°35′48.4″ E), Denmark. After harvesting the seaweed, it was immediately taken to the laboratory, rinsed with tap water to remove the sand and epiphytes attached to the seaweed, and immediately frozen in a −40 °C freezing room. The time from harvesting to freezing took approximately 2 h. The frozen seaweed was freeze-dried in a Christ Freeze Dryer Beta 1–8 (Osterode am Harz, Germany) for 2 days, then powdered with a Waring blender (Göteborg, Sweden), and sieved to remove large particles. The powdered seaweed was stored at −20 °C in a freezer, after being purged with nitrogen to avoid oxidation.

### 3.2. Pressurized Liquid Extractions

The extraction was performed using accelerated solvent extractor ASE (ASE 350, Dionex, Sunnyvale, CA, USA). For each experiment, 1 g of dried seaweed powder and 2 g of Ottawa sand were mixed and loaded into a 10 mL extraction cell equipped with a glass fiber filter on its bottom. Each experiment used a maximum operating pressure of 10 bar, 60% cell volume for rinsing, and purge time of 100 s. The extraction temperature was 120–200 °C, with a 10 °C increment. The extraction time was 5 min for all extraction conditions. The time needed for equilibration of the extraction conditions ranged from 6 to 9 min, so the total extraction time including equilibration time varied from 11 to 14 min. For each extraction, the condition the extraction was performed at was at least in duplicate. After completion of the extraction, the samples were stored at −20 °C in a freezer before characterization.

For the measurement of the extraction yield, an aliquot of each extract was transferred into falcon tubes, frozen overnight at −20 °C, and freeze-dried in a freeze dryer (Christ Freeze Dryer Beta 1–8, Osterode am Harz, Germany). Finally, the extraction yield was determined using the following equation:

$$\mathrm{Extraction}\;\mathrm{yield}=\frac{\mathrm{Mass}\;\mathrm{of}\;\mathrm{dried}\;\mathrm{extract}\;\left(g\right)\;}{\mathrm{Mass}\;\mathrm{of}\;\mathrm{Seaweed}\;\left(g\right)}\times 100$$

For comparison, conventional solvent extractions were also done with a modified method. One gram of the dried seaweed was submerged with 25 mL of 80% (*v*/*v*) ethanol in a water solution. This mixture was agitated on a shaker board (Heidolph Unimax 2010, Schwabach, Germany) at a speed of 150 rpm for 24 h in dark conditions and at room temperature. Afterward, the mixture was centrifuged (Sigma 4K15) at 2800 rpm for 10 min. The supernatant of this mixture was separated, and the extraction was repeated on the residue for an additional 24 h. Finally, the supernatants from the two extractions were pulled together and kept at −20 °C in a freezer until needed for further analysis.

### 3.3. Total Phenolic Compounds (TPC)

TPC was determined with a Folic-Ciocalteu assay, based on the method from Farvin and Jacobsen [40]. Briefly, 100 µL of the appropriately diluted extract was mixed with 750 µL of Folin–Ciocalteu reagent (10% *v*/*v* in water) in a 1.5 mL plastic microcuvette. After incubating the mixture for 5 min in the dark and at room temperature, 750 µL of sodium carbonate (Na_2_CO_3_, 7.5% *w*/*v* in water) was added. This solution was thoroughly mixed and incubated for 90 min more. Finally, the absorbance of this mixture was measured at 725 nm using a spectrophotometer (Shimadzu UV mini 1240, Duisburg, Germany). The TPC value of each extract was measured in triplicate and reported as a mean ± standard deviation. The TPC was calculated as milligrams of gallic acid equivalents (mg GAE) per 100 g of dry seaweed, based on a six-point standard curve of different gallic concentrations.

### 3.4. Phlorotannins Quantification (DMB Assay)

The phlorotannin content of the extracts was quantified using a method reported by Montero et al. [65]. Briefly, 50 µL of each diluted extract was mixed with a 250 µL DMBA reagent in a 96-well microplate. The DMBA reagent was prepared by mixing 2% (*w*/*v*) 2,4-methoxybenzaldhyde (DMBA) in glacial acetic acid and 6% (*v*/*v*) HCl in glacial acetic acid, just prior to use. The reaction mixture was incubated at room temperature in the dark for 60 min. Finally, the absorbance was read at 515 nm using a microplate reader BioTek Eon Microplate Spectrophotometer (Winooski, VT, USA). A calibration curve was made using phloroglucinol (0.98–62.5 µg/mL), and the result was reported as a microgram of phloroglucinol equivalent per gram of dried seaweed (µg PGE/g DW).

### 3.5. DPPH Radical Capacity Scavenging

For the determination of the DPPH radical scavenging capacity of the extracts, a method reported by Hermund et al. [54] was adopted. Briefly, an aliquot (100 µL) of extract at a concentration of 0.05–0.5 mg/mL was transferred into a 96-well microtiter plate. Then, 100 µL of DPPH (0.1 mM in ethanol) was added to each well. These mixtures were incubated for 30 min at room temperature in dark conditions. Afterwards, the absorbance of each mixture was measured at a wavelength of 517 nm in a BioTek Eon Microplate Spectrophotometer (Winooski, VT, USA). Effective concentrations for 50% inhibition (IC_50_) were calculated with Origin 2019b software, using a dose–response model.

### 3.6. Metal-Chelating Capacity

The method for measuring metal-chelating ability was based on Hermund et al. [54]. In brief, 100 μL of antioxidant extracts with concentrations of 0.5 to 10 mg/mL were pipetted into a 96-well microtiter plate, followed by the addition of 110 μL distilled water and 20 μL ferrous chloride solution (0.5 mM). After 3 min of incubation, 20 μL ferrozine solution (2.5 mM) was added to each well. The plate was incubated for 10 min in the dark at room temperature. Finally, the absorbance was measured at 562 nm in a BioTek Eon Microplate Spectrophotometer (Winooski, VT, USA). Effective concentrations for 50% chelating activity (IC_50_) were calculated with Origin 2019b software, using a dose–response model.

### 3.7. Phenolic Acid Identification

For the determination of individual phenolic acid in the extracts, a method reported by Farvin and Jacobsen [40] was used with minor modification. Briefly, the freeze-dried extracts were dissolved in deionized water at a concentration of 10 mg/mL. Aliquots of this were filtered using a syringe filter 0.22 µm before injection into an HPLC column (Prodigy 5 µm ODS-3 100 Å). The equipment consisted of an Agilent 1100 series HPLC (Agilent Technologies, Santa Clara, CA, USA) and a diode array detector (Agilent G1315A, Santa Clara, CA, USA).

Two types of mobile phases were used for this analysis: phosphoric acid in deionized water with a pH value of 3 (A) and a 1:1 mixture of methanol and acetonitrile (B). The gradient elution analysis program was as follows: 0–2 min, 5% (B); 2–20 min, increasing to 40% (B); 20–35 min, increasing to 100% (B); and 35–37 min, decreasing to 5% (B), with 3 min of post time at a flow rate of 0.9 mL/min. The injection volume of the sample was set at 20 µL, and the column was maintained at 25 °C. Detection with the diode array detector was done at four different wavelengths, i.e., 235, 255, 280, and 325 nm, with a reference wavelength of 360 nm. Quantification and identification of each phenolic acid were done by producing a calibration curve by injecting phenolic acid mixture standards with a concentration of each phenolic acid from 5.21 to 166.67 µg/mL. The limit of quantitation for all individual compounds, linearity range, and R^2^ is indicated in the supplementary file.

### 3.8. Isolation of Alginate and Fucoidan

The isolation of alginate and fucoidan was performed using the method described by [24] Briefly, 10 mL of each extract was mixed with an equal volume of CaCl_2_ (1% *w/w*) and kept in a refrigerator at 4 °C overnight. Then, the precipitated alginate was separated from the supernatant by centrifugation at 15,000× *g* for 15 min, followed by freeze-drying. To isolate the fucoidan, three volumes of absolute ethanol were added to the supernatant, which is obtained during the alginate precipitation step, and kept in the refrigerator at 4 °C overnight, followed by centrifugation. The pellet was first washed with ethanol and then with acetone, before it was freeze-dried. The alginate and fucoidan yields were calculated using the following equation:

$$\mathrm{Alginate}\;\mathrm{yield}=\;\frac{\mathrm{Mass}\;\mathrm{of}\;\mathrm{Crude}\;\mathrm{alginate}\;\left(g\right)}{\mathrm{Mass}\;\mathrm{of}\;\mathrm{Seaweed}\;\left(g\right)}\times 100$$

$$\mathrm{Crude}\;\mathrm{fucoidan}\;\mathrm{yield}=\frac{\mathrm{Mass}\;\mathrm{of}\;\mathrm{Crude}\;\mathrm{fucoidan}\;\left(g\right)}{\mathrm{Mass}\;\mathrm{of}\;\mathrm{Seaweed}\;\left(g\right)}\times 100$$

### 3.9. Statistical Analysis

The experimental values in this study were presented as ± deviation (SD). To determine the presence of significant difference in means of different treatments, a one-way ANOVA followed by a post hoc analysis by Tukey’s method were performed using statistical analysis software (Origin 2019b), where *p* < 0.5 was considered significant. The Pearson correlation analysis was also performed using the same software.

## 4. Conclusions

The marine environment is endowed with a large potential for the discovery of bioactive compounds. *Fucus vesiculosus* has a wide variety of bioactive compounds, which can be exploited for different purposes. To get the best out of this potential, the selection of efficient and green extraction methods plays a significant role. In this study, LPWE has shown a promising result for the extraction of phenolic compounds and polysaccharides. The extraction temperatures have shown a clear influence on the extraction yield, with the optimal being 200 °C for TPC, 130–150 °C for alginate, and 150–170 °C for fucoidan yield, and on the antioxidant activity of the extracts. By manipulating the extraction temperature, it is possible to selectively maximize the yield of targeted extracts. Moreover, the study showed that different antioxidant properties of the extract (DPPH radical scavenging (160 °C) or metalchelating activity (130 °C)) could selectively be obtained by manipulating the extraction temperature. The concentrations and bioactivity of this low polarity water-environment-friendly extraction showed as good/improved results, compared to the conventional solvent methods. However, further characterization and purification of the extracts are required to better understand the nature of the extracts, and how this influences antioxidant activity.

## Figures and Tables

**Table 1:** DPPH and radical scavenging and metal-chelating (MC) activity of the *F. vesiculosus* extracts at different extraction temperatures.

Extraction Temperature (°C)	DPPH IC_50_ (mg/mL)	MC IC_50_ (mg/mL)
120	0.23 ± 0.08 ^a^	2.45 ± 0.03 ^cd^
130	0.19 ± 0.00 ^ab^	1.39 ± 0.04 ^d^
140	0.19 ± 0.08 ^ab^	1.50 ± 0.06 ^d^
150	0.20 ± 0.01 ^ab^	1.56 ± 0.11 ^d^
160	0.14 ± 0.02 ^b^	1.69 ± 0.15 ^d^
170	0.18 ± 0.02 ^ab^	4.55 ± 0,19 ^cd^
180	0.17 ± 0.04 ^b^	6.35 ± 0.40 ^c^
190	0.14 ± 0.02 ^b^	12.09 ± 1.23 ^b^
200	0.17 ± 0.03 ^ab^	23.05 ± 3.79 ^a^

Note: Values are mean ± SD (*n* = 3), and values followed by different letters in the same column are significantly different (*p* < 0.05).

## Supplementary Materials

The following supporting information can be downloaded at: https://www.mdpi.com/article/10.3390/md20040263/s1, Figures: Chromatograms for all tasted phenolic acids and standards; Table S1: The R^2^, limit of quantification, and linearity range all tested phenolic acids.
