# Supplementary material for: Effect of Extraction Temperature on Pressurized Liquid Extraction of Bioactive Compounds from Fucus vesiculosus
Source: Mar Drugs. 2022 Apr 12;20(4):263. doi: 10.3390/md20040263 (PMC9028048; doi:10.3390/md20040263)
Supplement: Supplementary file 1 [file marinedrugs-20-00263-s001.zip › marinedrugs-1654019-supplementary.pdf]

Chromatograms for all tasted phenolic acids and standards

Standard

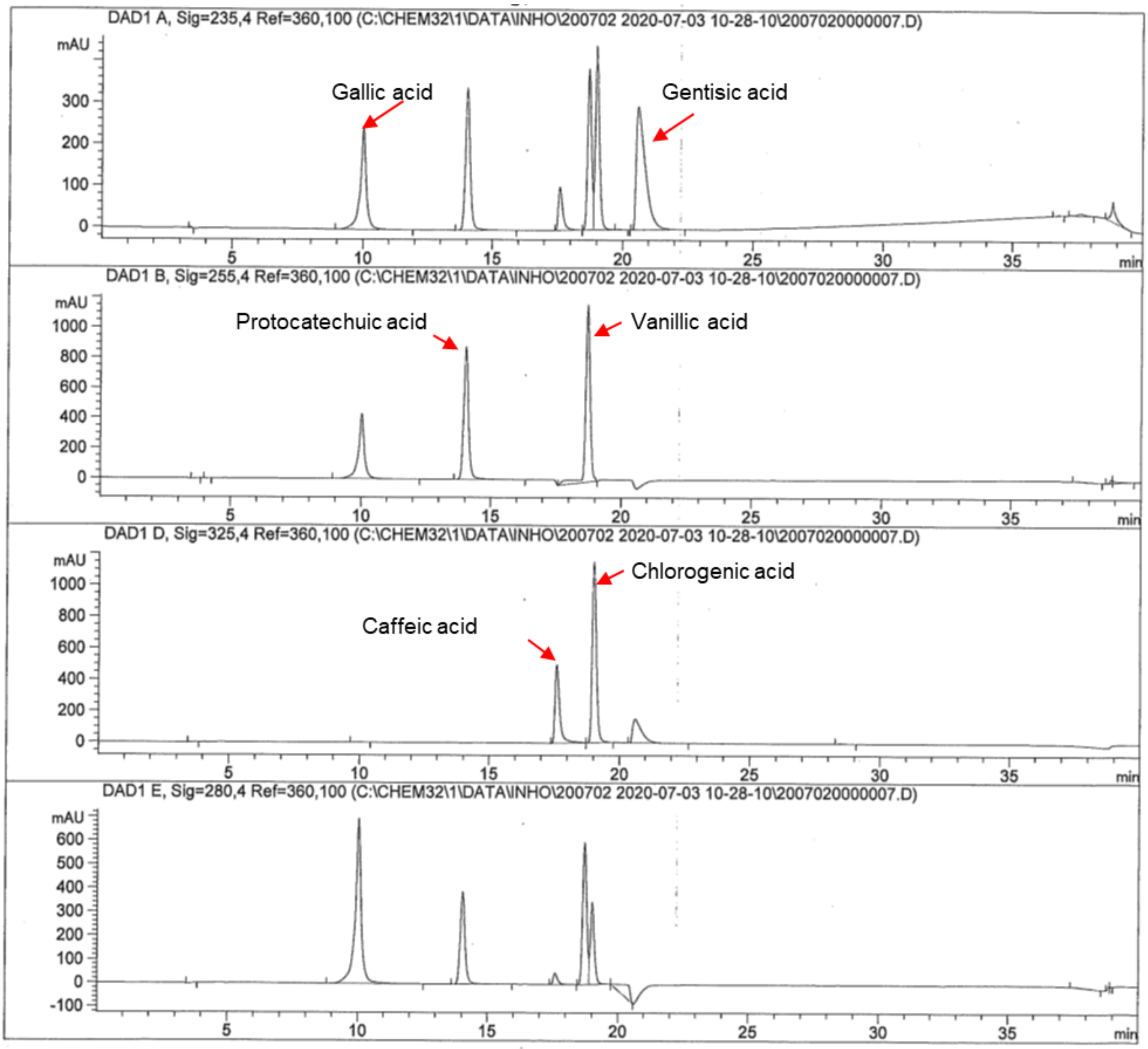

HPLC chromatogram of phenolic acid in *F. vesiculosus* extracted at 120°C

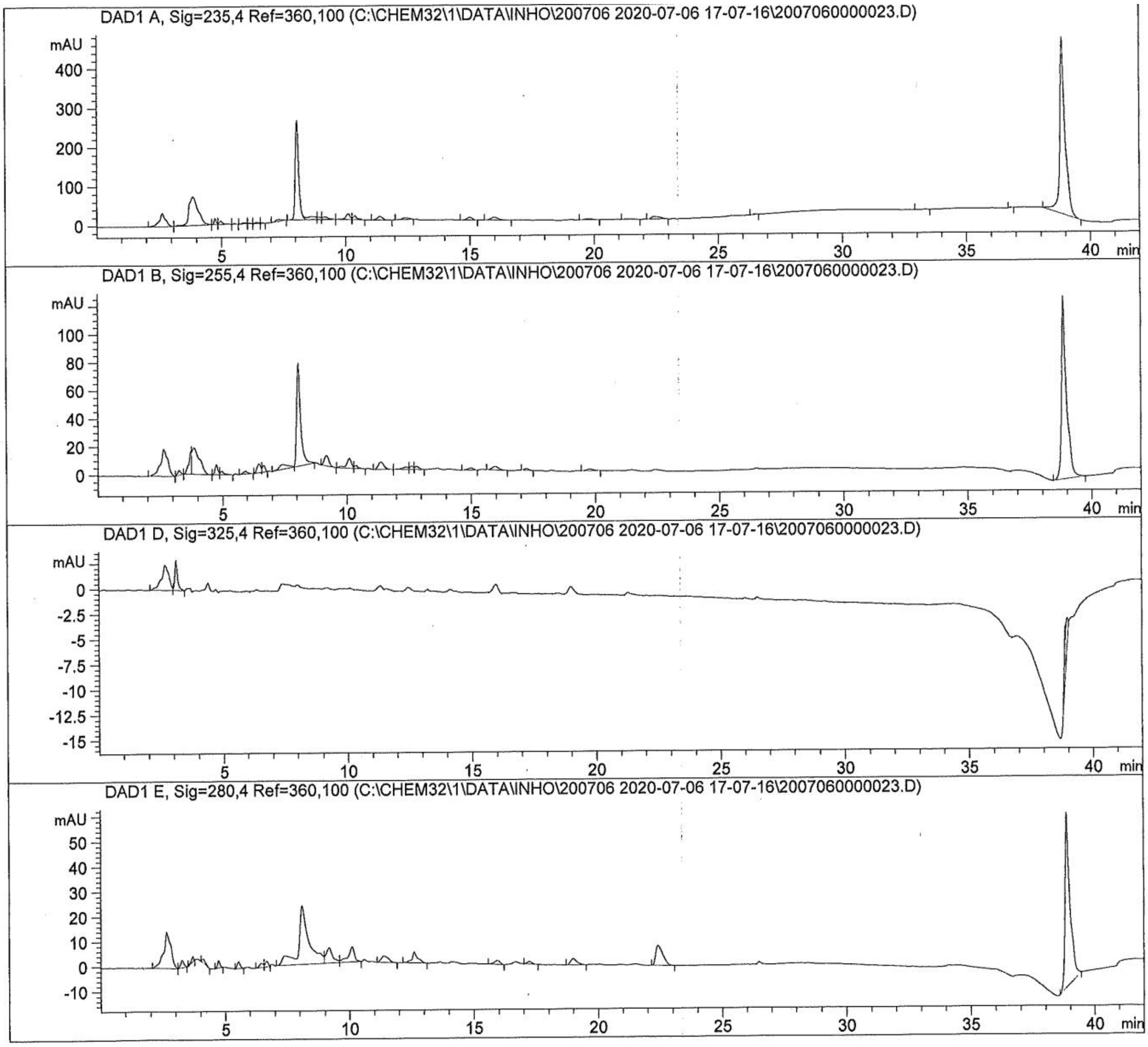

HPLC chromatogram of phenolic acid in *F. vesiculosus* extracted 130°C

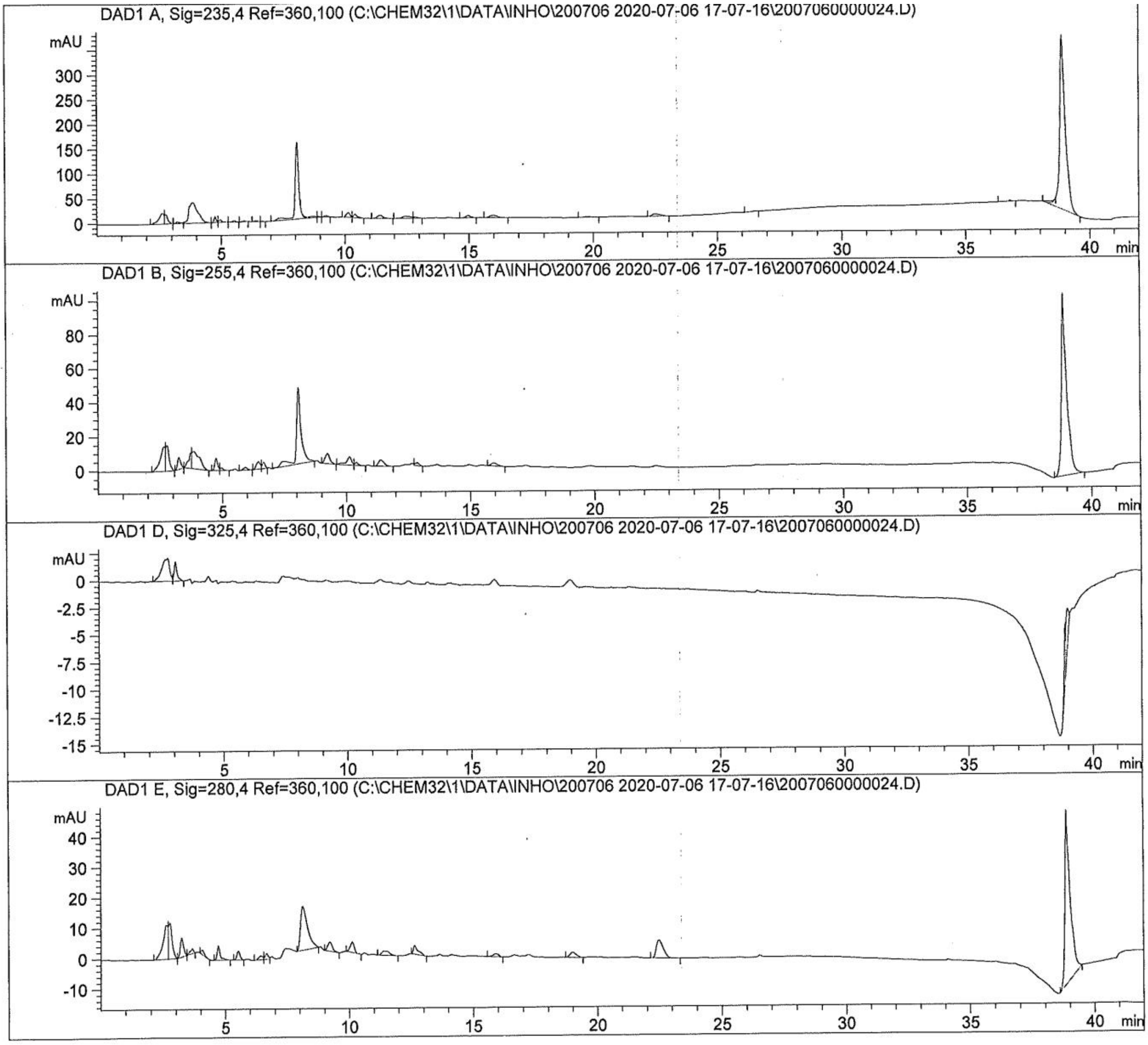

HPLC chromatogram of phenolic acid in *F. vesiculosus* extracted 140°C

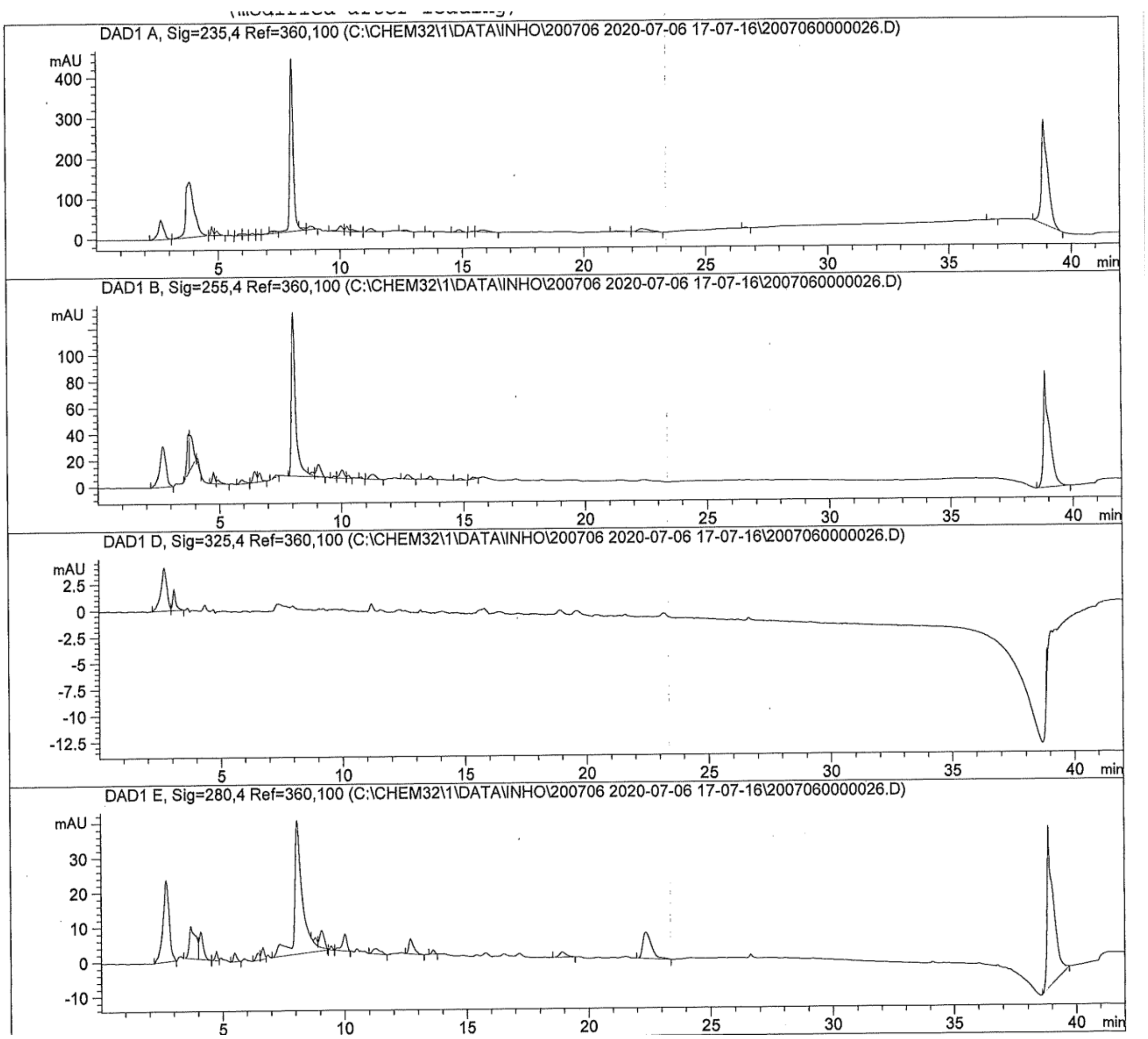

HPLC chromatogram of phenolic acid in *F. vesiculosus* extracted 150°C

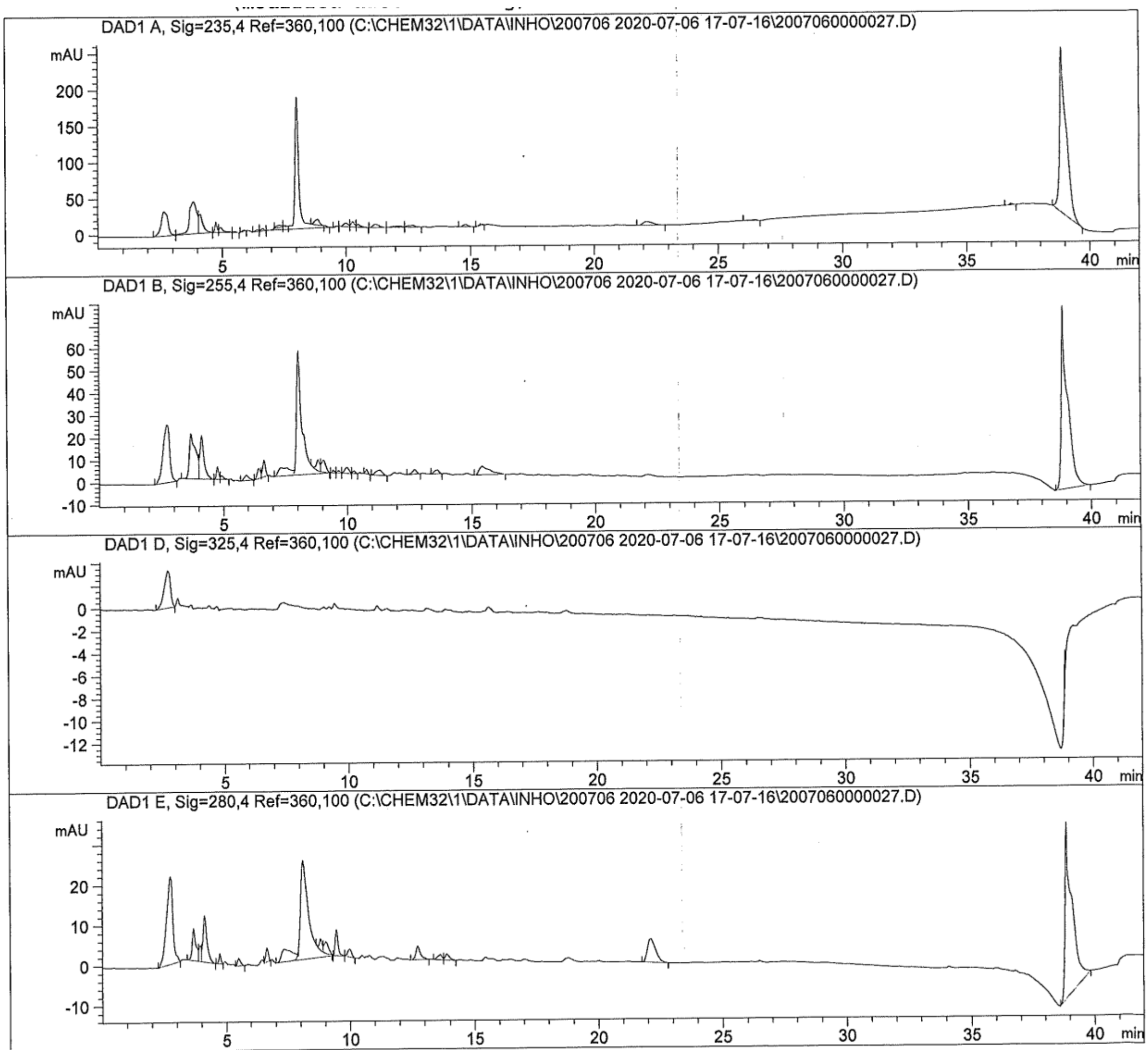

HPLC chromatogram of phenolic acid in *F. vesiculosus* extracted 160°C

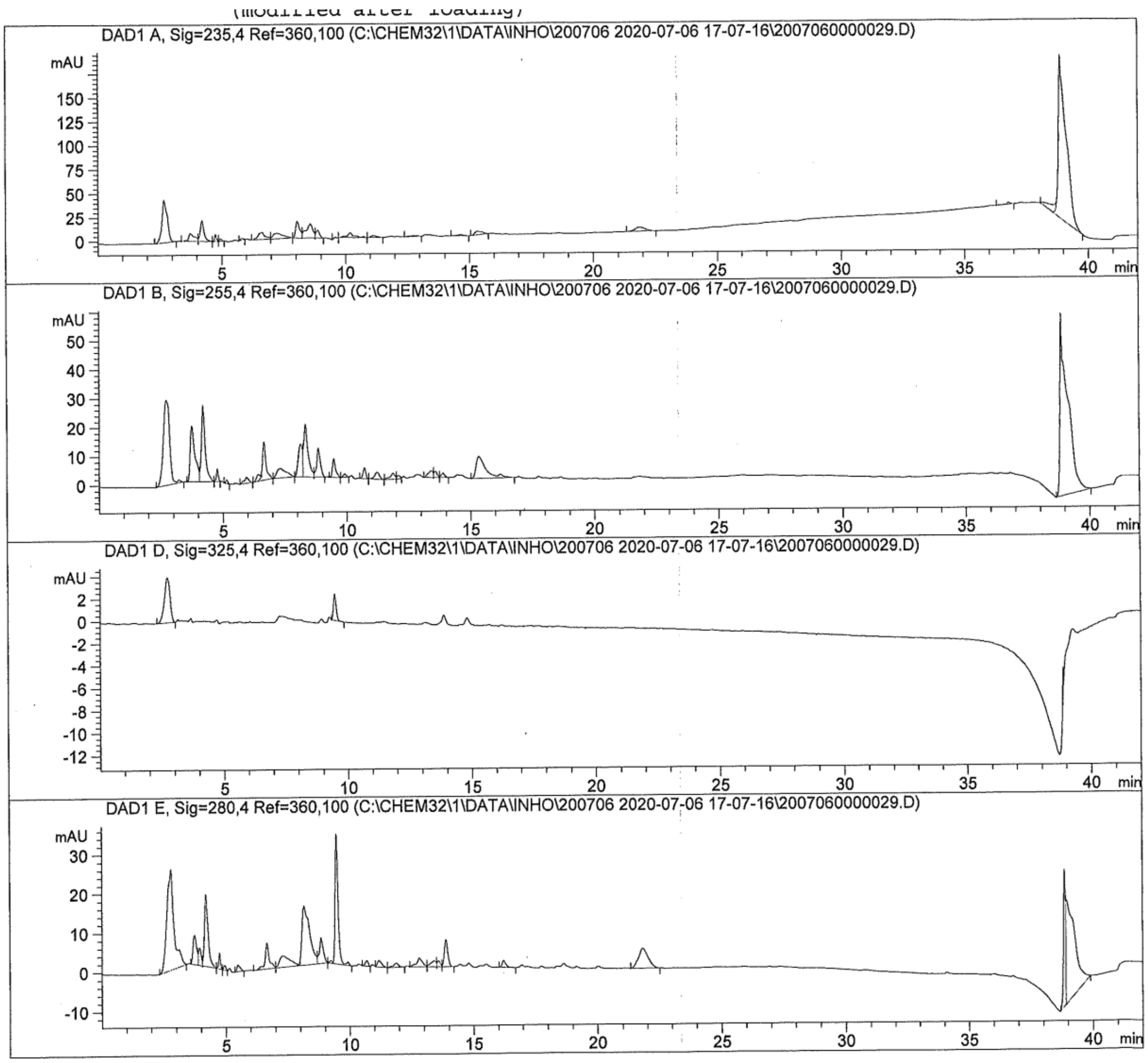

HPLC chromatogram of phenolic acid in *F. vesiculosus* extracted 170°C

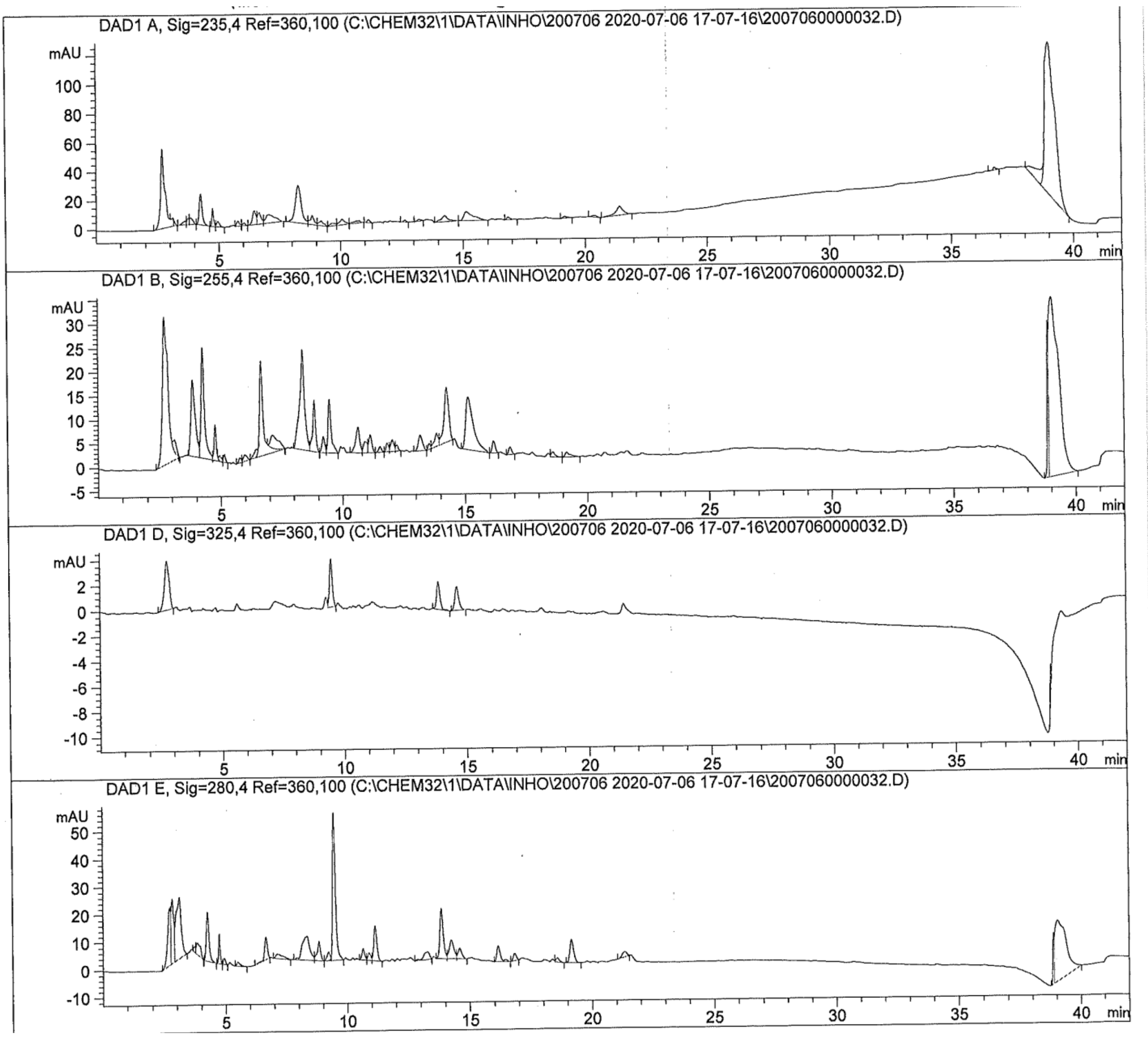

HPLC chromatogram of phenolic acid in *F. vesiculosus* extracted 180°C

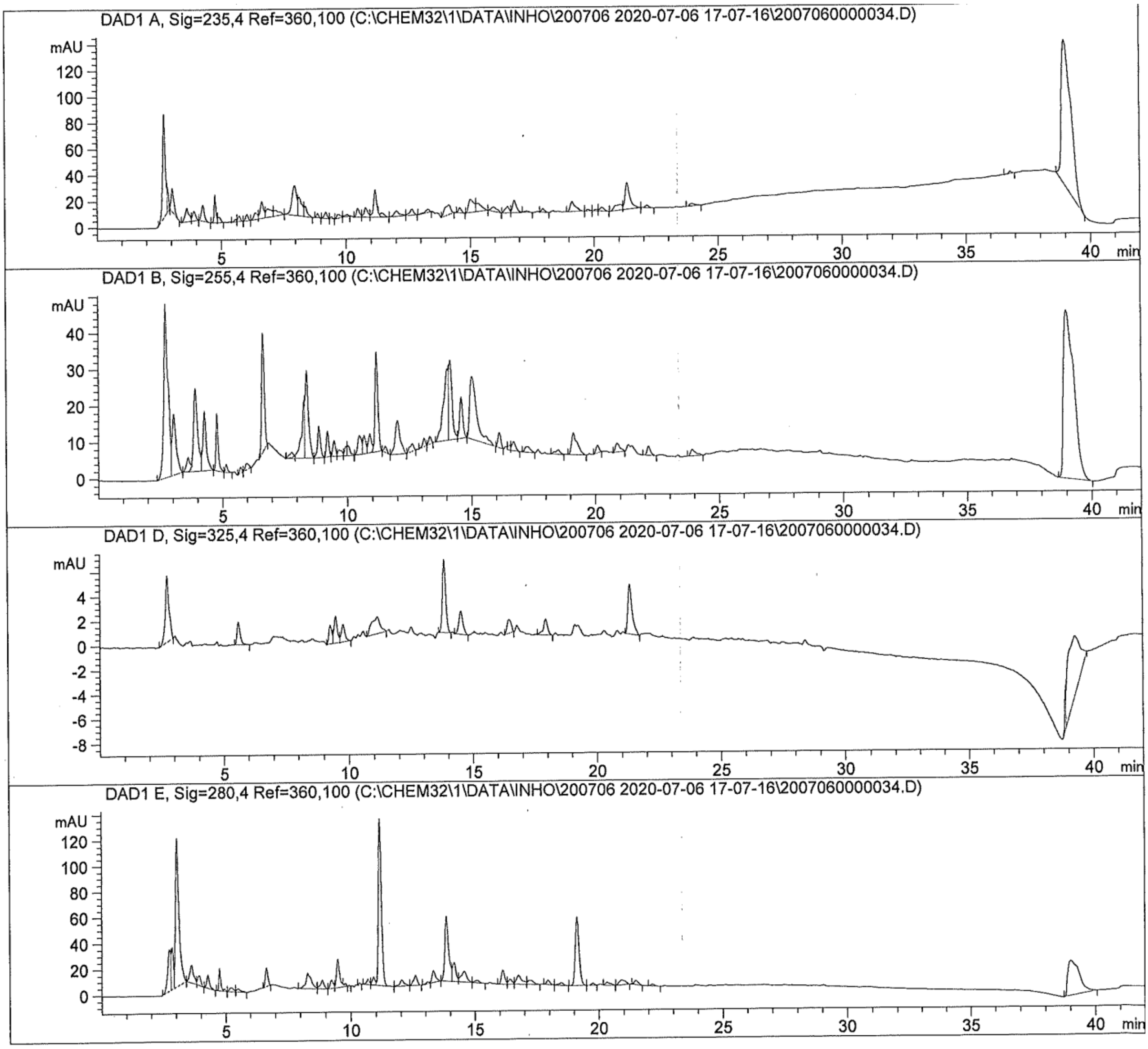

HPLC chromatogram of phenolic acid in *F. vesiculosus* extracted 190°C

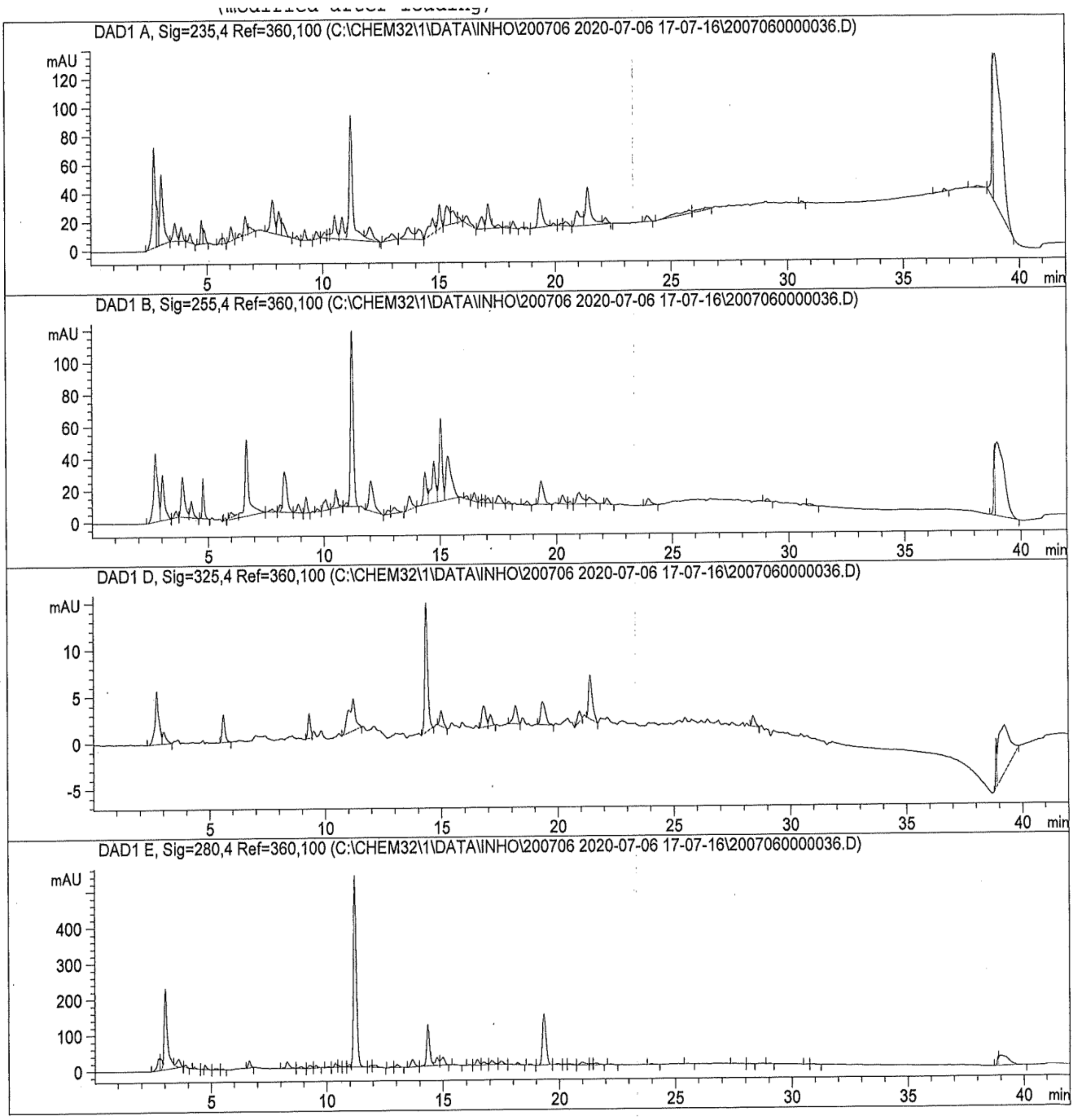

HPLC chromatogram of phenolic acid in *F. vesiculosus* extracted 200°C

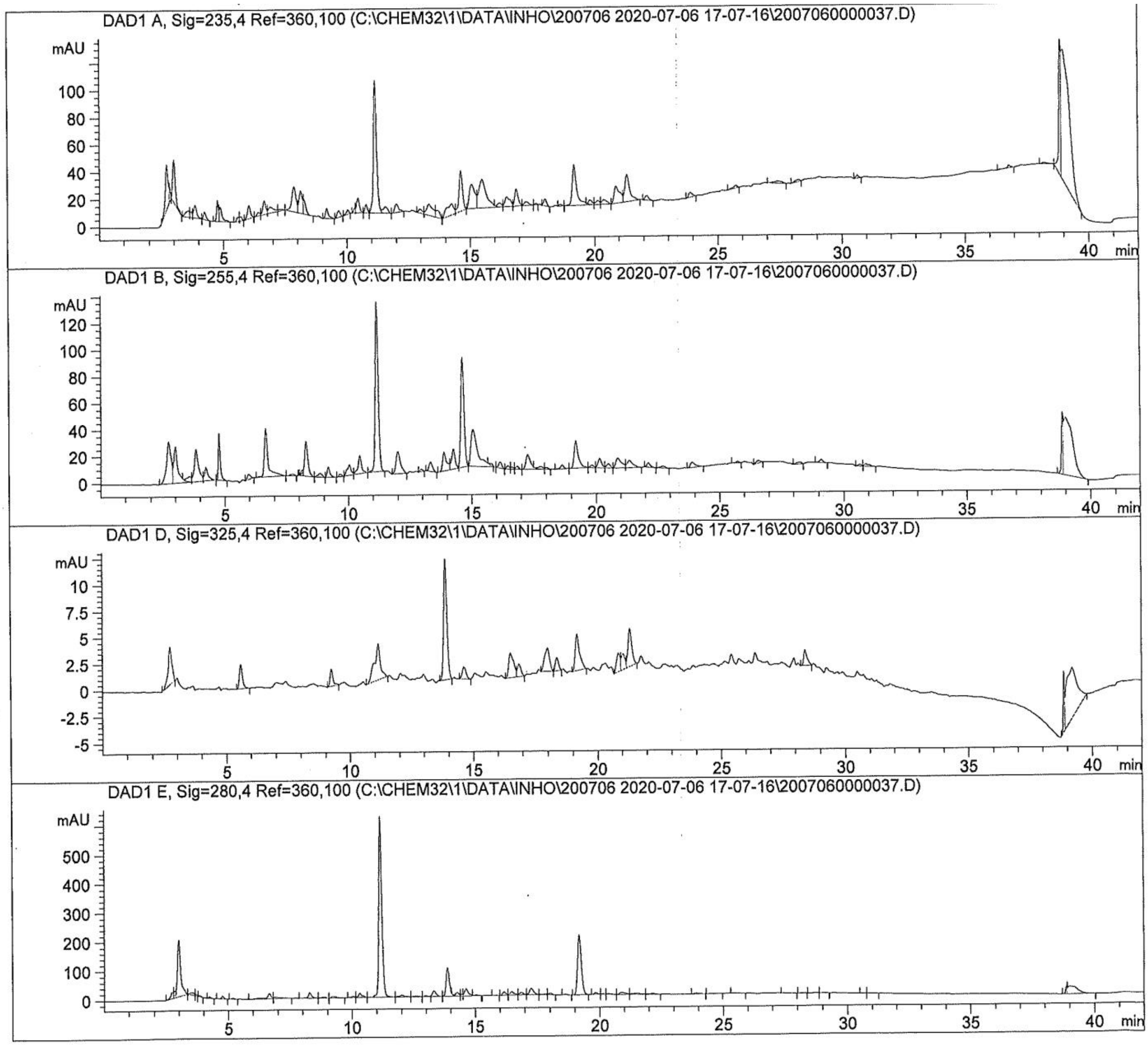

Table S1: The R^2, limit of quantification, and linearity range all tested phenolic acids.

Compounds	R^2	LOQ	Linearity range (µg/mg)
Gallic acid	0.0998	32.81	5.21-166.67
Protocatechuic acid	0.9990	30.18	5.21-166.67
Chlorogenic acid	0.9987	34.94	5.21-166.67
Vanillic acid	0.9995	20.96	5.21-166.67
Caffeic acid	0.9996	19.52	5.21-166.67
Gentisic acid	0.9979	43.73	5.21-166.67
